# Supplementary material for: SOXC are critical regulators of adult bone mass
Source: Nat Commun. 2024 Apr 5;15:2956. doi: 10.1038/s41467-024-47413-2 (PMC10997656; doi:10.1038/s41467-024-47413-2)
Supplement: Supplementary file 1 — Supplementary Info [file 41467_2024_47413_MOESM1_ESM.pdf]

SUPPLEMENTARY INFORMATION

SOXC are critical regulators of adult bone mass

Marco Angelozzi,* Anirudha Karvande, and Véronique Lefebvre*

Department of Surgery, Division of Orthopaedics, Children's Hospital of Philadelphia, Pennsylvania, USA

This document contains:

- **Supplementary figures 1 to 11**
- **Supplementary tables 1 and 2**

SUPPLEMENTARY FIGURES

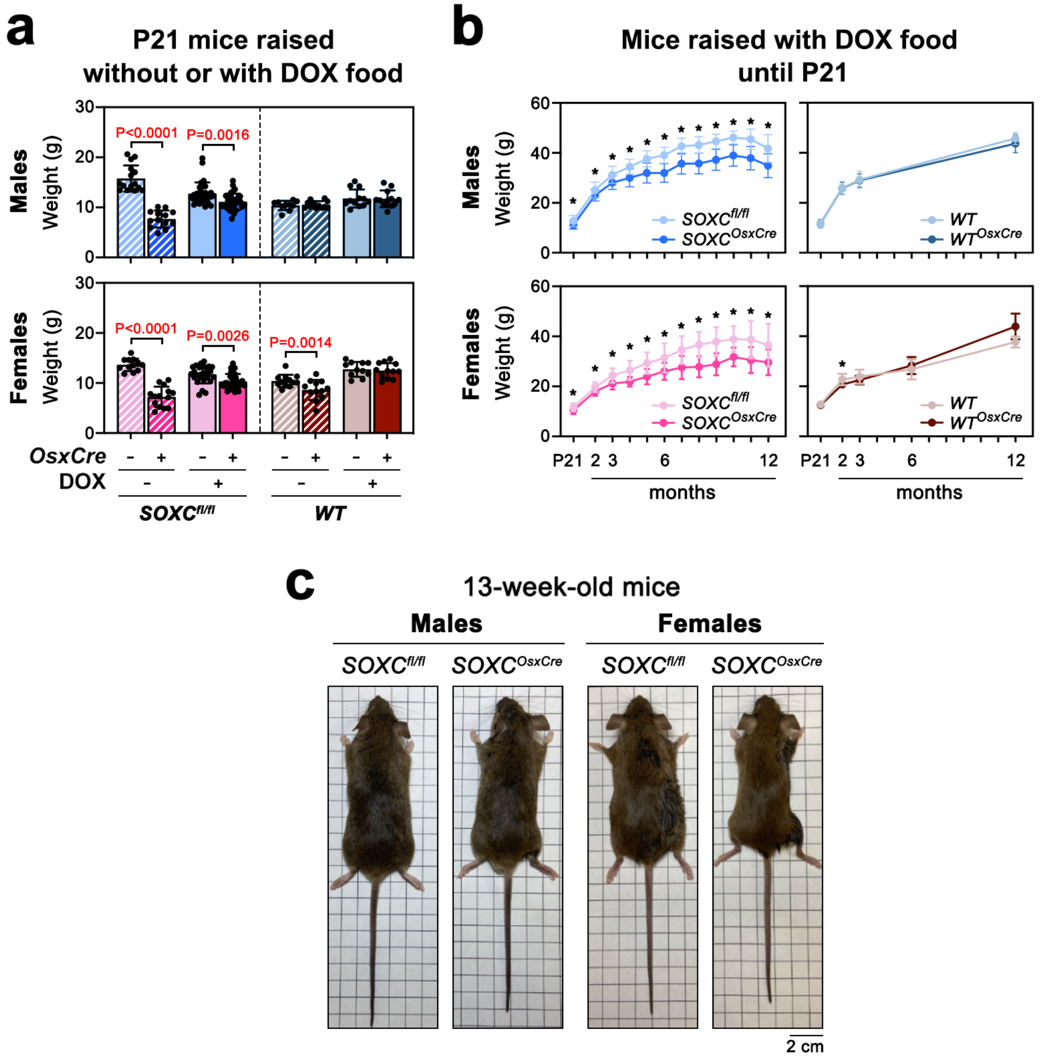

Supplementary figure 1. External phenotype of *SOXC*^{OsxCre} mice

a. Weights of 3-week-old mice raised since conception in cages containing food pellets supplemented or not with doxycycline (DOX food). Each dot represents one mouse. Bars and brackets represent means and standard deviations, respectively. Statistical differences were assessed by two-sided unpaired Student's t-tests. P-values ≤0.05 are shown.

b. Growth curves of *WT*^{OsxCre}, *SOXC*^{OsxCre} and control males and females conceived and raised in cages with DOX food. Statistical differences were assessed by two-sided unpaired Student's t-tests. Asterisks point to significant differences (p ≤0.05) in weights between experimental groups.

c. Representative pictures of 13-week-old *SOXC*^{OsxCre} and control males and females conceived and raised in cages with DOX food.

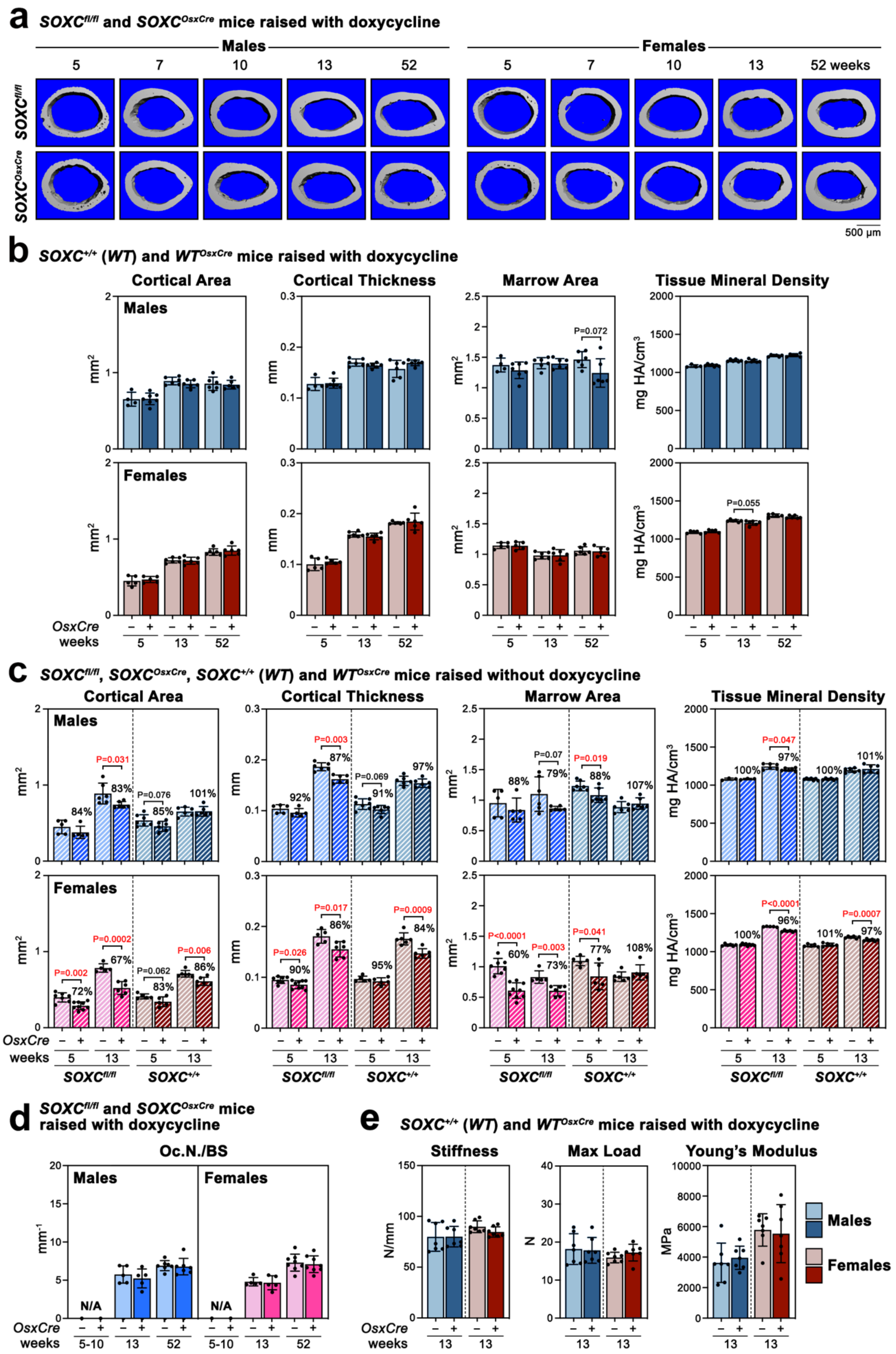
a
SOXCfl/fl and SOXCOsxCre mice raised with doxycycline
Males
Females
5
7
10
13
52
52 weeks
SOXCfl/fl
SOXCOsxCre
500 µm
b
SOXC+/+ (WT) and WTOsxCre mice raised with doxycycline
Cortical Area
Cortical Thickness
Marrow Area
Tissue Mineral Density
Males
Females
mm2
mm
mg HA/cm3
P=0.072
P=0.055
OsxCre
weeks
c
SOXCfl/fl, SOXCOsxCre, SOXC+/+ (WT) and WTOsxCre mice raised without doxycycline
Cortical Area
Cortical Thickness
Marrow Area
Tissue Mineral Density
Males
Females
P=0.031
P=0.076
P=0.003
P=0.069
P=0.07
P=0.019
P=0.047
P=0.002
P=0.0002
P=0.062
P=0.006
P=0.026
P=0.017
P=0.0009
P<0.0001
P=0.003
P=0.041
P<0.0001
P=0.0007
SOXCfl/fl
SOXC+/+
d
SOXCfl/fl and SOXCOsxCre mice raised with doxycycline
Oc.N./BS
Males
Females
mm-1
N/A
5-10
e
SOXC+/+ (WT) and WTOsxCre mice raised with doxycycline
Stiffness
Max Load
Young's Modulus
N/mm
N
MPa
Males
Females

Supplementary figure 2. Cortical bone phenotype of femurs from *WT*^{OsxCre} and *SOXC*^{OsxCre} mice

a. Representative µCT images of femur cortices from *SOXC*^{OsxCre} and control males and females at 5 to 52 weeks.

b. Quantification of cortical bone area, cortical thickness, marrow area, and cortical bone mineral density of femurs from *WT*^{OsxCre} and control males and females at 5, 13 and 52 weeks conceived and raised until weaning with DOX food. Each dot corresponds to a distinct mouse. Bars and brackets represent means and standard deviations, respectively. Statistical analysis was performed by two-sided unpaired Student's t-tests. No significant difference was detected between *WT*^{OsxCre} and control mice for any parameter, although p-values were near the significance threshold (0.05) for two parameters, as indicated.

c. Quantification of cortical bone parameters of femurs from *SOXC*^{OsxCre}, *WT*^{OsxCre} and control males and females at 5 and 13 weeks conceived and raised without DOX food. The percentages of data obtained for *WT*^{OsxCre} versus control mice are indicated. Statistical analysis was performed by two-sided unpaired Student's t-tests. P-values near (black) and below 0.05 (red) are shown.

d. Static histomorphometry of osteoclast numbers (Oc.N./BS) per bone surface at the endosteal surfaces of femur cortices from *SOXC*^{OsxCre} and control males and females at 13 and 52 weeks. Surfaces of mice between 5 and 10 weeks of age had virtually no osteoclasts (N/A = not available). Statistical analysis was performed by two-sided unpaired Student's t-tests, but no significant difference was detected between *SOXC*^{OsxCre} and control mice.

e. Biomechanical properties of femurs from *WT*^{OsxCre} and control males and females at 13 weeks determined in three-point bending tests. Statistical analysis was performed by two-sided unpaired Student's t-tests, but no significant difference was detected between *WT*^{OsxCre} and control mice for any parameter.

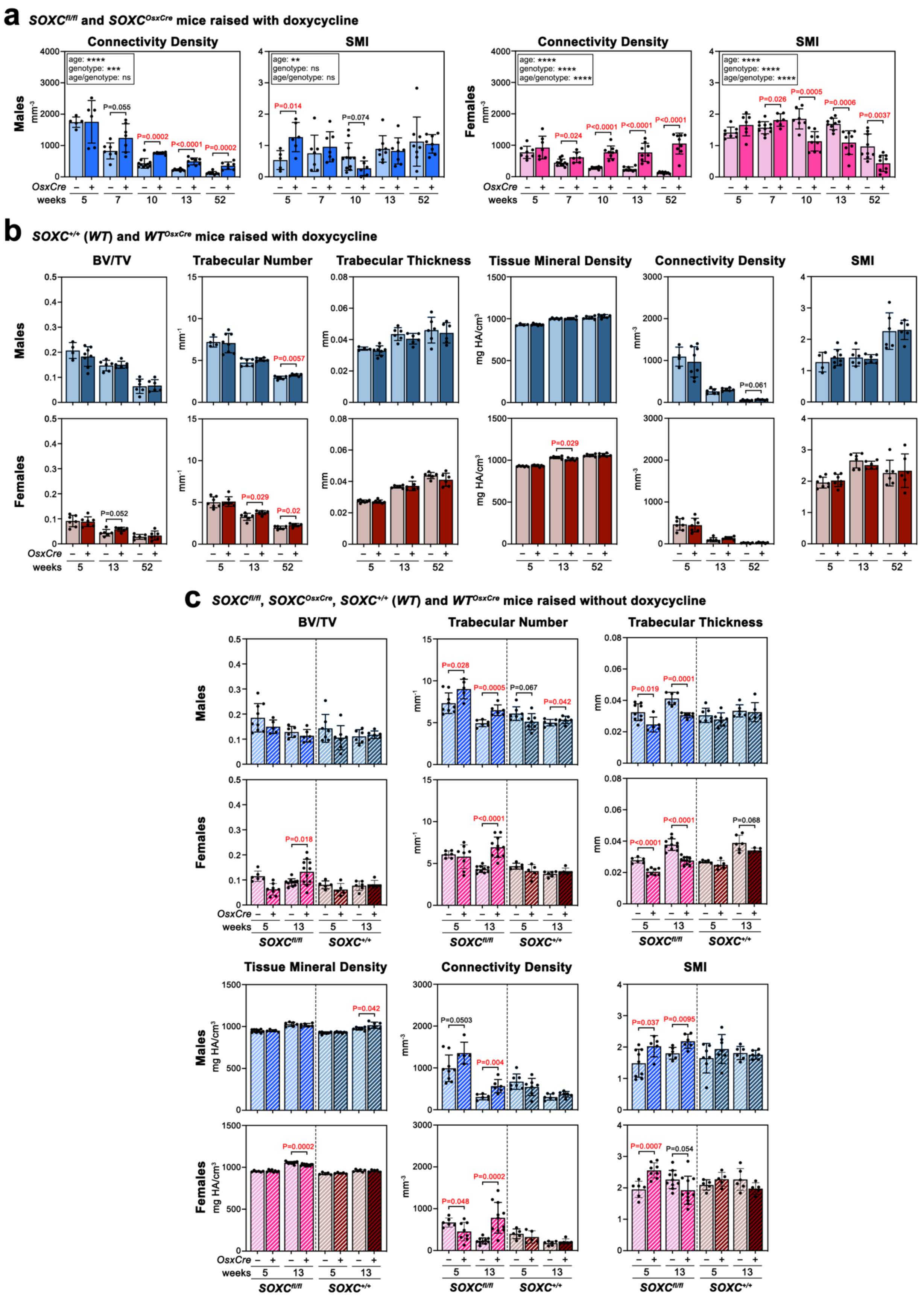
a SOXCfl/fl and SOXCOsxCre mice raised with doxycycline
Connectivity Density
SMI
Males
Females
age: ****
genotype: ***
age/genotype: ns
age: **
genotype: ns
age/genotype: ns
age: ****
genotype: ****
age/genotype: ****
P=0.055
P=0.0002
P<0.0001
P=0.0002
P=0.014
P=0.074
P=0.024
P<0.0001
P<0.0001
P<0.0001
P=0.026
P=0.0005
P=0.0006
P=0.0037
OsxCre
weeks
b SOXC+/+ (WT) and WTOsxCre mice raised with doxycycline
BV/TV
Trabecular Number
Trabecular Thickness
Tissue Mineral Density
Connectivity Density
SMI
mm-1
mm
mg HA/cm3
mm-3
P=0.0057
P=0.061
P=0.052
P=0.029
P=0.02
P=0.029
c SOXCfl/fl, SOXCOsxCre, SOXC+/+ (WT) and WTOsxCre mice raised without doxycycline
P=0.028
P=0.0005
P=0.067
P=0.042
P=0.019
P=0.0001
P=0.018
P<0.0001
P<0.0001
P<0.0001
P=0.068
P=0.0503
P=0.004
P=0.037
P=0.0095
P=0.0002
P=0.048
P=0.0002
P=0.0007
P=0.054
SOXCfl/fl
SOXC+/+

Supplementary figure 3. Trabecular bone phenotype of femurs from *WT*^{OsxCre} and *SOXC*^{OsxCre} mice

a. µCT quantification of connectivity density and structural model index (SMI) for trabecular bone in femurs from *SOXC*^{OsxCre} and control males and females at 5 to 52 weeks. Mice were conceived and raised until weaning with DOX food. Each dot corresponds to a distinct mouse. Bars and brackets represent means and standard deviations, respectively. Statistical differences were assessed by two-sided unpaired Student's t-tests. P-values lower (red) and near (black) 0.05 are indicated. Box, statistical differences obtained using a two-way ANOVA test of the effects of age and genotype and their interaction (ns, non-significant; *, p ≤0.05; **, p ≤0.01; ***, p ≤0.001; ****, p ≤0.0001).

b. µCT quantification of trabecular bone parameters in femurs from *WT*^{OsxCre} and control mice at 5, 13 and 52 weeks of age. Mice were conceived and raised until weaning with DOX food. Data are presented as described in (a). Of note, *WT*^{OsxCre} females showed a mild increase in trabecular number at 13 and 52 weeks. This increase was less marked than in *SOXC*^{OsxCre} mice (1.2 *vs* 1.5-fold at 13 weeks, p = 0.006; and 1.2 *vs* 2.4-fold at 52 weeks, p = 0.0002).

c. µCT quantification of trabecular bone parameters in femurs from *SOXC*^{OsxCre}, *WT*^{OsxCre} and control mice at 5 and 13 weeks of age. Mice were conceived and raised until weaning without DOX food. Data are presented as described in (a).

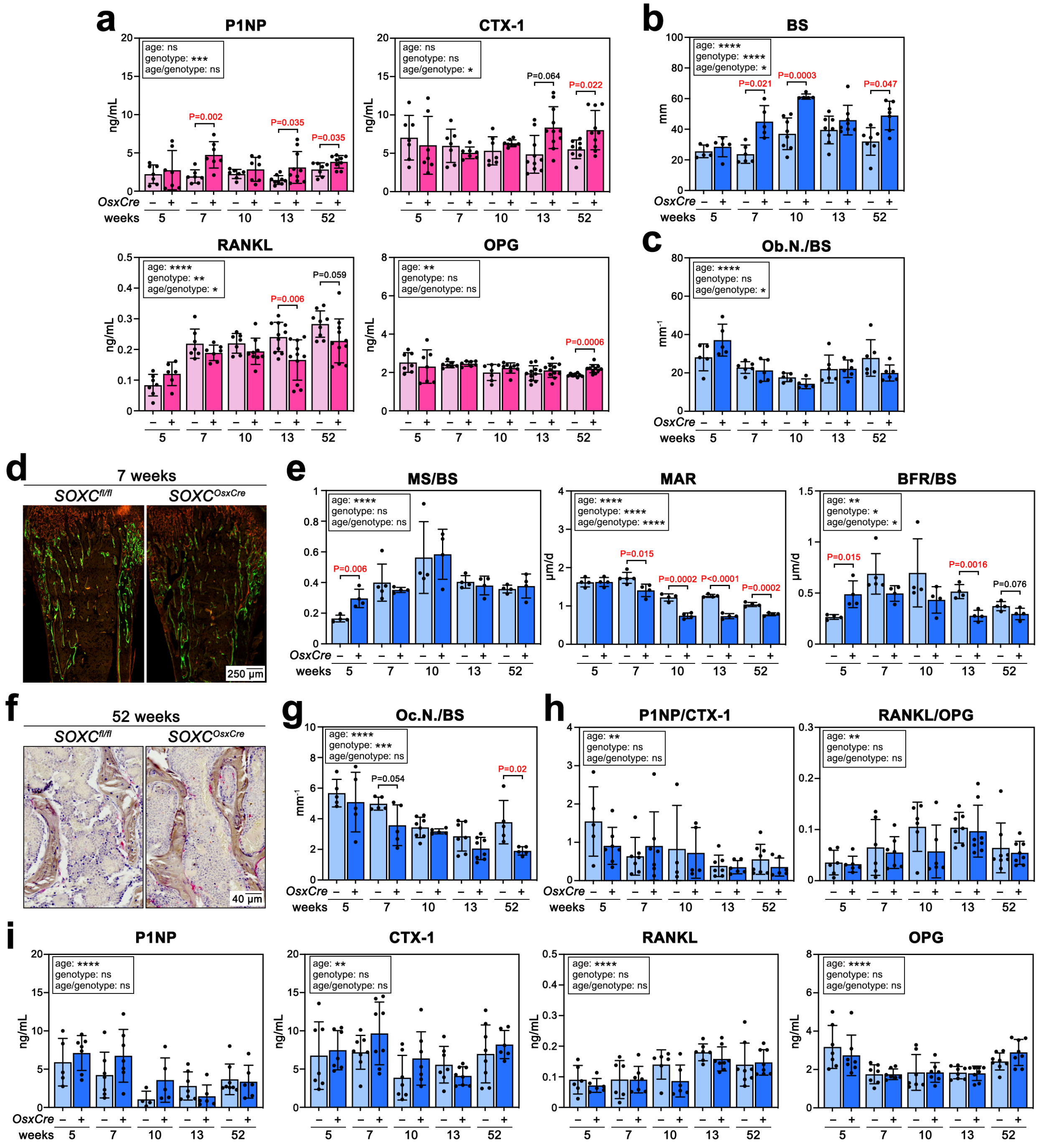

Supplementary figure 4. Femur histomorphometry and serum analyses for *SOXC*^{OsxCre} mice

a. Serum levels of P1NP, CTX-1, RANKL and OPG quantified by ELISA in *SoxC*^{OsxCre} and control females at 5 to 52 weeks. Each dot corresponds to a distinct mouse. Bars and brackets represent means and standard deviations, respectively. Statistical differences were assessed by two-sided unpaired Student's t-tests. P-values lower (red) and near (black) 0.05 are indicated. Box, statistical differences obtained using a two-way ANOVA test of the effects of

age and genotype and their interaction (ns, non-significant; *, p ≤0.05; **, p ≤0.01; ***, p ≤0.001; ****, p ≤0.0001).

b. Static histomorphometry of the trabecular bone surface (BS) of femurs from *SOXC*^{OsxCre} and control males at 5 to 52 weeks. Data are presented as described in (a).

c. Static histomorphometry of osteoblast numbers per bone surface (Ob.N./BS) for *SOXC*^{OsxCre} and control males at 5 to 52 weeks. Data are presented as described in (a). No significant differences were detected between age-matched control and mutant groups.

d. Representative pictures of *in vivo* labeling of newly synthesized mineralized matrix in trabecular bones from 7-week-old *SOXC*^{OsxCre} and control males. Mice were injected with calcein (green, changed to white with Adobe Photoshop) and alizarin red (red) nine and two days before euthanasia, respectively. The assay was repeated for all the mice analyzed in (e).

e. Dynamic histomorphometry of the mineralizing surface (MS/BS), mineral apposition rate (MAR) and bone formation rate (BFR/BS) per bone surface of femurs from *SOXC*^{OsxCre} and control males at 5 to 52 weeks. Data are presented as described in (a).

f. Representative pictures of TRAP staining of sections through trabecular bone and bone marrow of femurs from 52-week-old *SOXC*^{OsxCre} and control males. TRAP^{+} cells, red; bone matrix, light brown. The assay was repeated for all the mice analyzed in (g).

g. Static histomorphometry of osteoclast numbers (Oc.N./BS) per bone surface for *SOXC*^{OsxCre} and control males at 5 to 52 weeks. Data are presented as described in (a).

h. Serum level ratios of P1NP/CTX-1 (bone formation/resorption indicators) and RANKL/OPG (osteoclastogenesis activator/inhibitor) in *SOXC*^{OsxCre} and control males at 5 to 52 weeks. Data are presented as described in (a). No significant differences were detected between age-matched control and mutant groups.

i. Serum levels of P1NP, CTX-1, RANKL and OPG quantified by ELISA in *SoxC*^{OsxCre} and control males at 5 to 52 weeks. Data are presented as described in (a). No significant differences were detected between age-matched control and mutant groups.

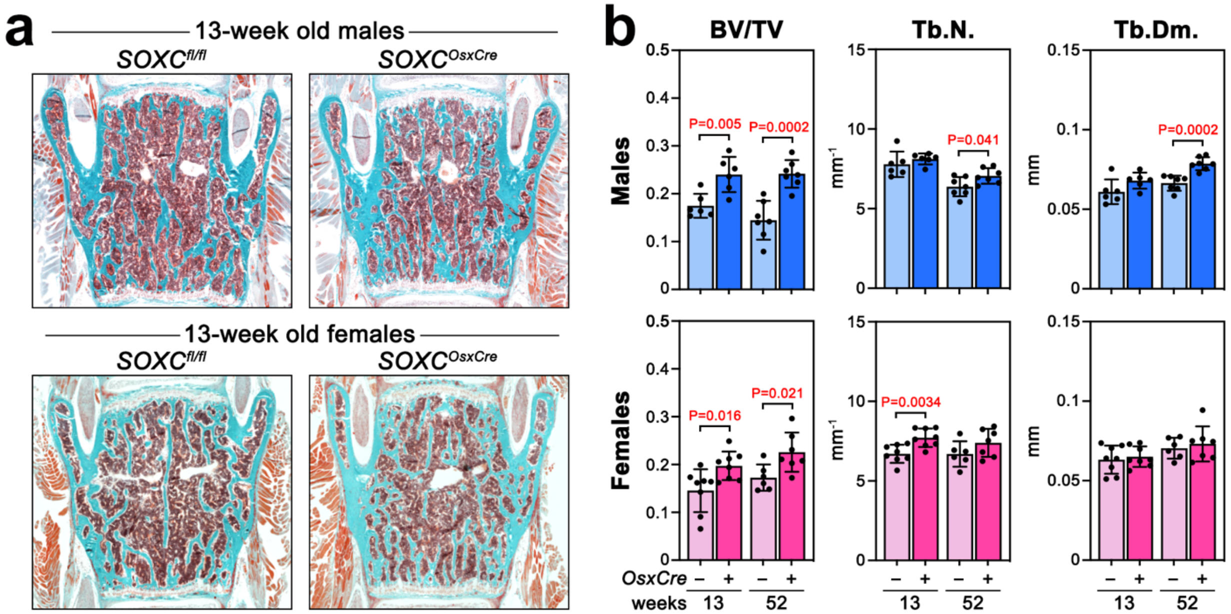

Supplementary figure 5. Trabecular bone phenotype of vertebrae from *SOXC*^{OsxCre} mice

a. Representative pictures of Masson-Goldner's trichrome stained sections through the L3 vertebral body of 13-week-old *SOXC*^{OsxCre} and control males and females. Mice were conceived and raised until weaning with DOX food. They belonged to the same cohort as those for which femurs were analyzed (Figs. 1 and 2). Mineralized bone, green; osteoid (not mineralized) tissue and bone marrow, pink/dark red; red blood cells, bright red. The assay was repeated for all the mice analyzed in (b).

b. Quantification by histomorphometry of BV/TV, trabecular number, and trabecular diameter (Tb. Dm.) in vertebra from 13-week-old *SOXC*^{OsxCre} and control males and females. Each dot corresponds to a distinct mouse. Bars and brackets represent means and standard deviations, respectively. Statistical differences were assessed by two-sided unpaired Student's t-tests. P-values ≤0.05 are indicated.

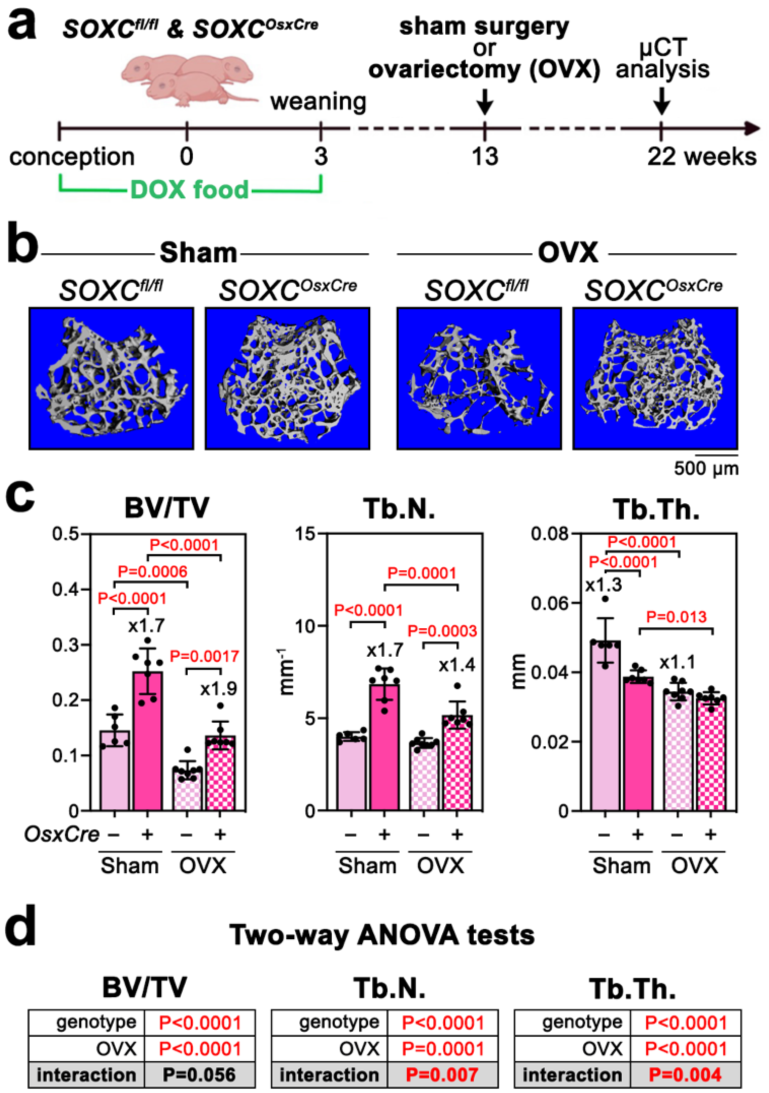

Supplementary figure 6. Effect of ovariectomy on the trabecular bone phenotype of femurs from *SOXC*^{OsxCre} females

a. Experimental design for the analysis of females subjected to ovariectomy or sham surgery (image created with BioRender.com).

b. Representative µCT images of the secondary spongiosa of femurs from sham-operated and ovariectomized *SOXC*^{OsxCre} and control females.

c. Quantification of BV/TV, trabecular number, and thickness of femurs from sham-operated and ovariectomized *SOXC*^{OsxCre} and control females. Each dot corresponds to a distinct mouse. Bars and brackets represent means and standard deviations, respectively. Parameter fold changes between control and mutant groups are indicated. Statistical differences were assessed by one-way ANOVA tests followed by Tukey's multiple comparisons test. P-values ≤0.05 are reported.

d. Results from two-way ANOVA tests performed to assess the effects of genotype, surgery, and their interactions on trabecular bone properties in *SOXC*^{OsxCre} and control mice. Data used for this test are presented in panel c.

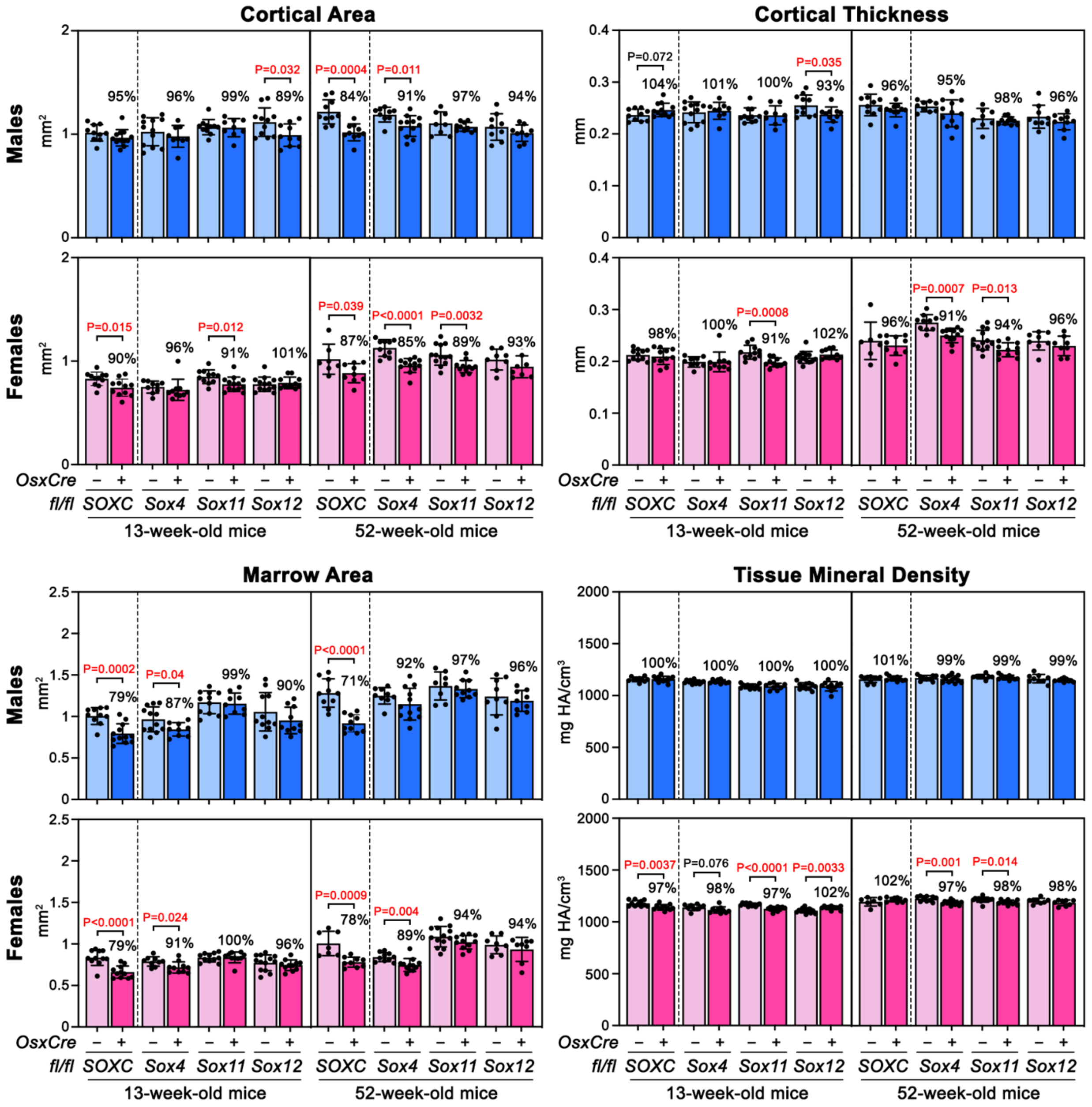

Supplementary figure 7. Cortical bone phenotype of SOXC single mutant mice

Quantification of cortical bone area, cortical thickness, marrow area, and cortical bone mineral density of femurs from *SOXC*[OsxCre], *Sox4*[OsxCre], *Sox11*[OsxCre], *Sox12*[OsxCre] and control males and females at 13 and 52 weeks. Each dot corresponds to a distinct mouse. Bars and brackets represent means and standard deviations, respectively. The percentages of mutant versus control average values are indicated. Statistical differences were assessed by two-sided unpaired Student's t-tests. P-values near 0.05 (black) and below 0.05 (red) are indicated.

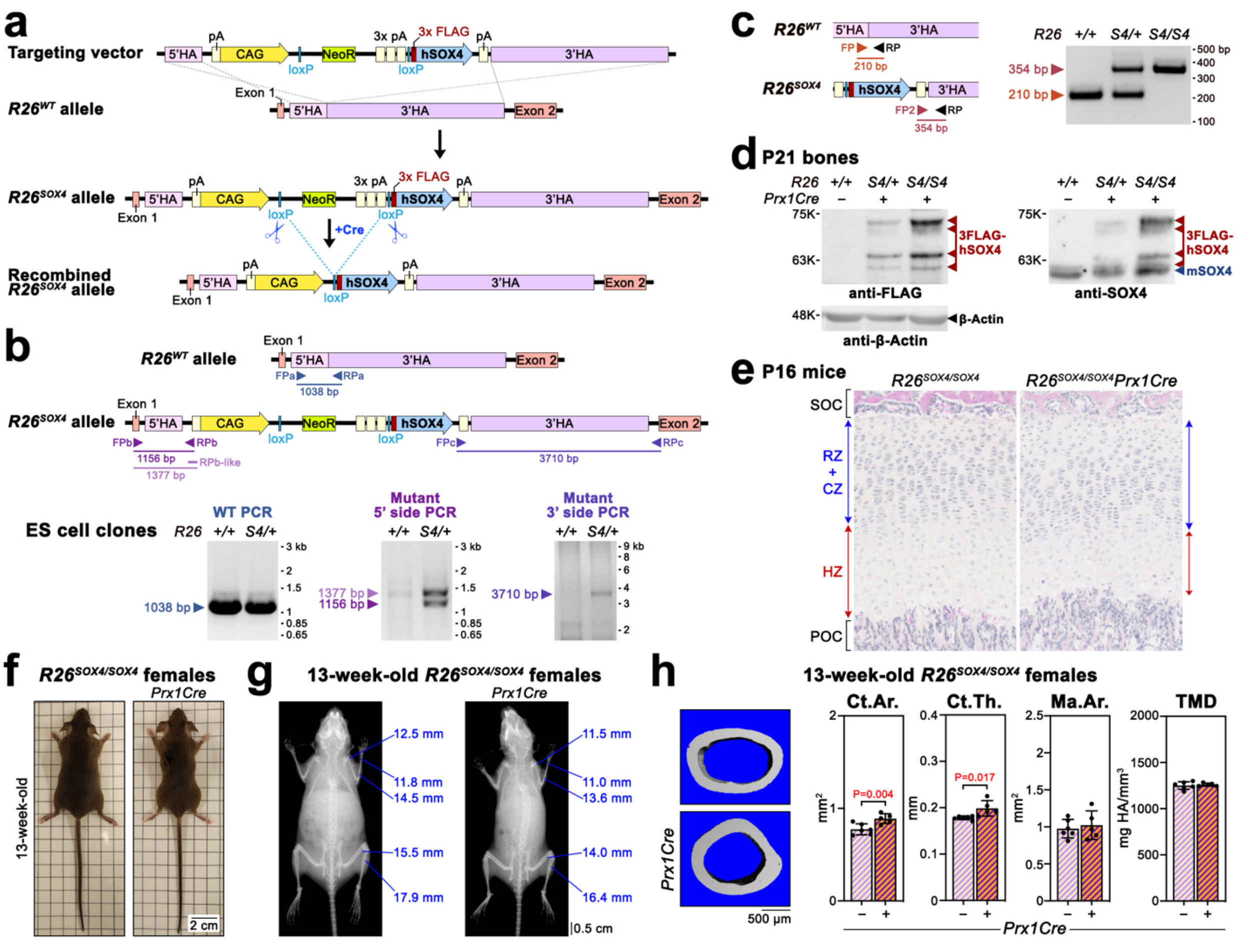

Supplementary figure 8. Generation and analysis of mice harboring *R26*^{SOX4} allele(s)

a. Gene knock-in strategy to generate a *R26*^{SOX4} allele in the mouse. A targeting vector was constructed that contained 5' and 3' homology arms (HAs) corresponding to adjacent sequences in the *R26* first intron. Knock-in sequences introduced between the two arms contained pA (polyadenylation site), CAG (cytomegalovirus early enhancer, chicken β-actin promoter, first exon and intron, and rabbit β-globin splice acceptor), loxP sites flanking *NeoR* (neomycin resistance cassette) followed by three pAs, the coding sequence of the human SOX4 protein tagged at the N-terminus with a three-FLAG epitope and linker sequence, and a pA. DNA homologous recombination (dotted crosses) of the wild-type allele (*R26*^{WT}) with this targeting vector created *R26*^{SOX4} allele. Recombination of this allele by Cre recombinase resulted in excision of *NeoR* permitting conditional expression of 3FLAG-hSOX4.

b. Identification of embryonic stem cell clones having undergone DNA homologous recombination by PCR. Three pairs of PCR primers were designed. The first pair comprised a forward primer (FPa) annealing in the 5' arm of homology and a reverse primer (RPa) annealing in the 3' arm of homology. It amplified a 1038-bp sequence in the *R26*^{WT} allele. The second pair of primers comprised a forward primer (FPb) annealing 5' of the 5' HA and a reverse primer (RPb) annealing in the 5' end of the knock-in sequence. It amplified sequences of 1156 bp and 1377 bp. The longer product resulted from annealing of RPb with a sequence (RPb-like) that only had one mismatch compared to that matching the primer sequence. The

third pair of primers comprised a forward primer (FPc) annealing in the 3' end of the knock-in sequence and a reverse primer (RPc) annealing 3' of the 3' HA. It amplified a 3710-bp sequence. The last two PCRs allowed to identify clones with a properly created $R26^{SOX4}$ allele. Images show PCR products obtained for $R26^{WT}$ (+/+) and $R26^{SOX4/+}$ (*S4/+*) clones following DNA electrophoresis in agarose gels. 48 cell clones were screened by PCR.

c. PCR genotyping of $R26^{+/+}$, $R26^{SOX4/+}$ and $R26^{SOX4/SOX4}$ mice. The $R26^{WT}$ allele was identified through amplification of a 210-bp sequence with forward (FP) and reverse (RP) primers annealing on either side of the two HAs. The $R26^{SOX4}$ allele was identified through amplification of a 354-bp sequence with a forward primer (FP) annealing in the 3' end of the knock-in sequence and the same reverse primer as for the $R26^{WT}$ PCR. Genotyping was performed using all three primers in the same PCR. The image shows PCR products obtained for $R26^{+/+}$ (+/+), $R26^{SOX4/+}$ (*S4/+*) and $R26^{SOX4/SOX4}$ (*S4/S4*) mice following DNA electrophoresis in an agarose gel. PCR genotyping is performed on every experimental mouse.

d. Western blot detection of the mSOX4 and 3FLAG-hSOX4proteins in bone extracts from P21 $R26^{+/+}$, $R26^{SOX4/+}$ and $R26^{SOX4/SOX4}$ *Prx1Cre* mice. Blots were hybridized with anti-FLAG, SOX4, and β-actin antibodies. Note that the 3FLAG-SOX4 protein appears to be produced in several forms, possibly resulting from differential post-translational events. Also, note that the amount of 3FLAG-SOX4 protein is similar to that of endogenous mSOX4 protein. The assay was repeated for bone extracts from two independent samples.

e. Representative images of proximal tibia growth plates from $R26^{SOX4/SOX4}$ and $R26^{SOX4/SOX4}$ *Prx1Cre* female littermates at P16. SOC, secondary ossification center. RZ + CZ, reserve and columnar zones. HZ, hypertrophic zone. POC, primary ossification center. Note that the hypertrophic zone is shorter in the mutant growth plate, possibly due to accelerated turnover of cartilage into endochondral bone. The assay was repeated on sections from two independent samples.

f. Representative pictures of 13-week-old $R26^{SOX4/SOX4}$ and $R26^{SOX4/SOX4}$ *Prx1Cre* female littermates. Note that the mutant is slightly smaller than its control sister.

g. Faxitron images of the skeleton of the same mice as in panel f. Note that long bones are shorter in the mutant than in its control sister. The assay was repeated on two mice.

h. µCT analysis of femur cortical bone in 13-week-old $R26^{SOX4/SOX4}$ and $R26^{SOX4/SOX4}$ *Prx1Cre* females. Left, representative images of bone cortices. Right, quantification of cortical area, cortical thickness, marrow area, and tissue mineral density. Each dot corresponds to a distinct mouse. Bars and brackets represent means and standard deviations, respectively. Statistical differences were assessed by two-sided unpaired Student's t-tests. P-values ≤0.05 are indicated.

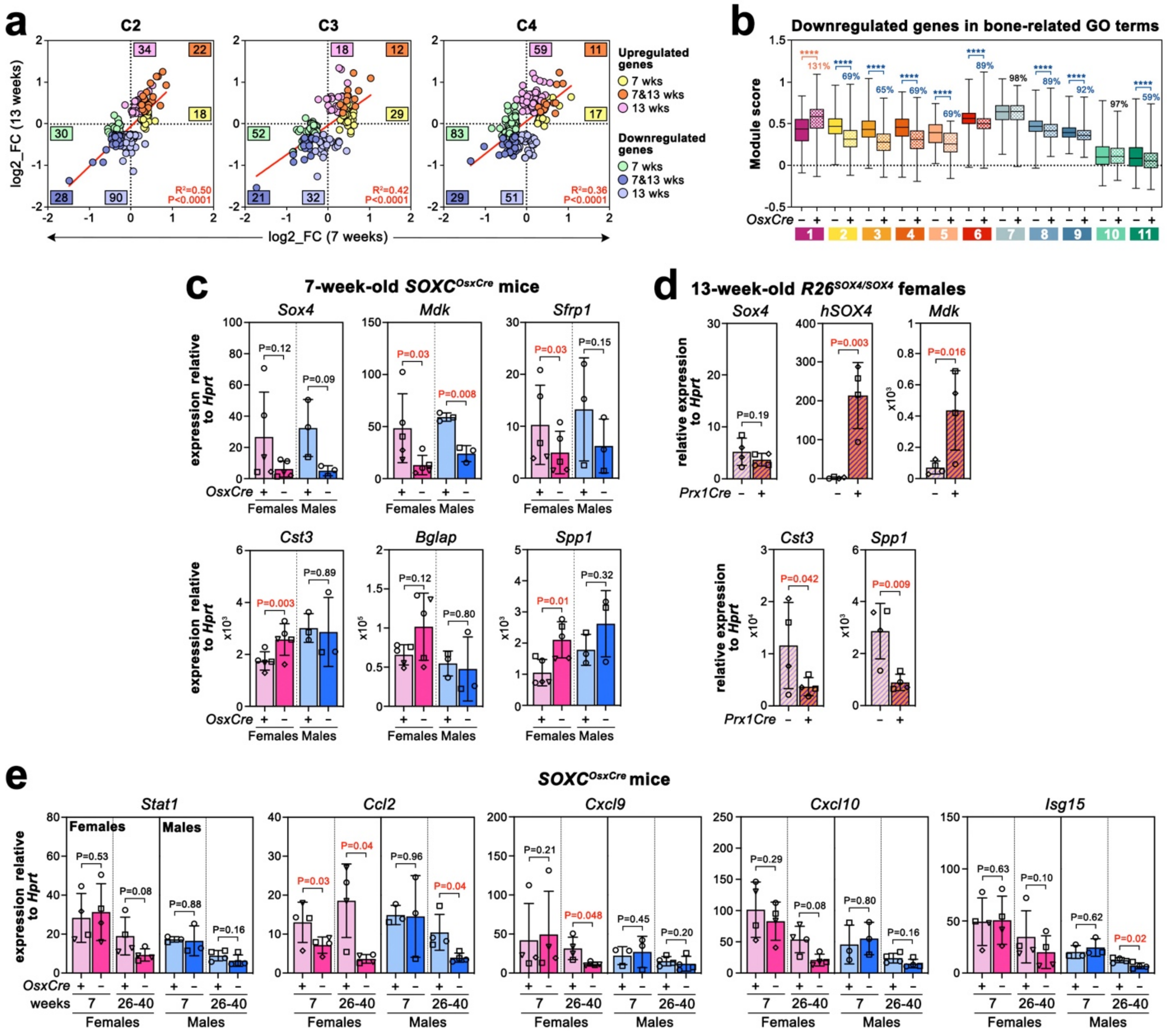

Supplementary figure 9. Transcriptomic analysis of *SOXC*^{OsxCre} C2-C4 clusters

a. Dot plots comparing the expression fold-changes of DEGs detected in the mutant versus control C2, C3 and C4 clusters at 7 and 13 weeks. Yellow and light greed dots, genes affected only at 7 weeks; pink and light blue dots, genes affected only at 13 weeks; orange and blue dots, genes affected at both ages. The linear correlation existing for the fold-changes between the two ages is shown with a red line, and its significance is indicated by its p-value and coefficient of determination (R^2).

b. Histogram plots of module scores generated using the downregulated genes in the C2-C4 mutant clusters that match bone-related GO terms. The module score difference obtained between control and mutant populations is indicated as a percentage and its significance, assessed using a Mann-Whitney test, is also indicated (****, p <0.0001).

c. RT-qPCR assay of the expression levels of *Sox4* and selected C2-C4 DEGs matching bone-related GO terms in non-hematopoietic bone and marrow cell suspensions obtained as described for scRNA-seq assays but using different 7-week-old *SOXC*^{OsxCre} and control males

and females. Each dot corresponds to a distinct mouse. Same symbols indicate littermates. Bars and brackets represent means and standard deviations, respectively. Statistical differences were assessed by two-sided paired Student's t-tests. P-values near 0.05 (black) and below 0.05 (red) are indicated.

d. RT-qPCR assay performed and presented as described in (c) but using cells from 13-week-old $R26^{SOX4/SOX4}$*Prx1Cre* and control females.

e. RT-qPCR assay of the expression levels of selected C2-C4 DEGs matching interferon-related GO terms in non-hematopoietic bone and marrow cell suspensions obtained as described for scRNA-seq assays but using different 7- and 26-to-40-week-old $SOXC^{OsxCre}$ and control males and females. Data are presented as in (c).

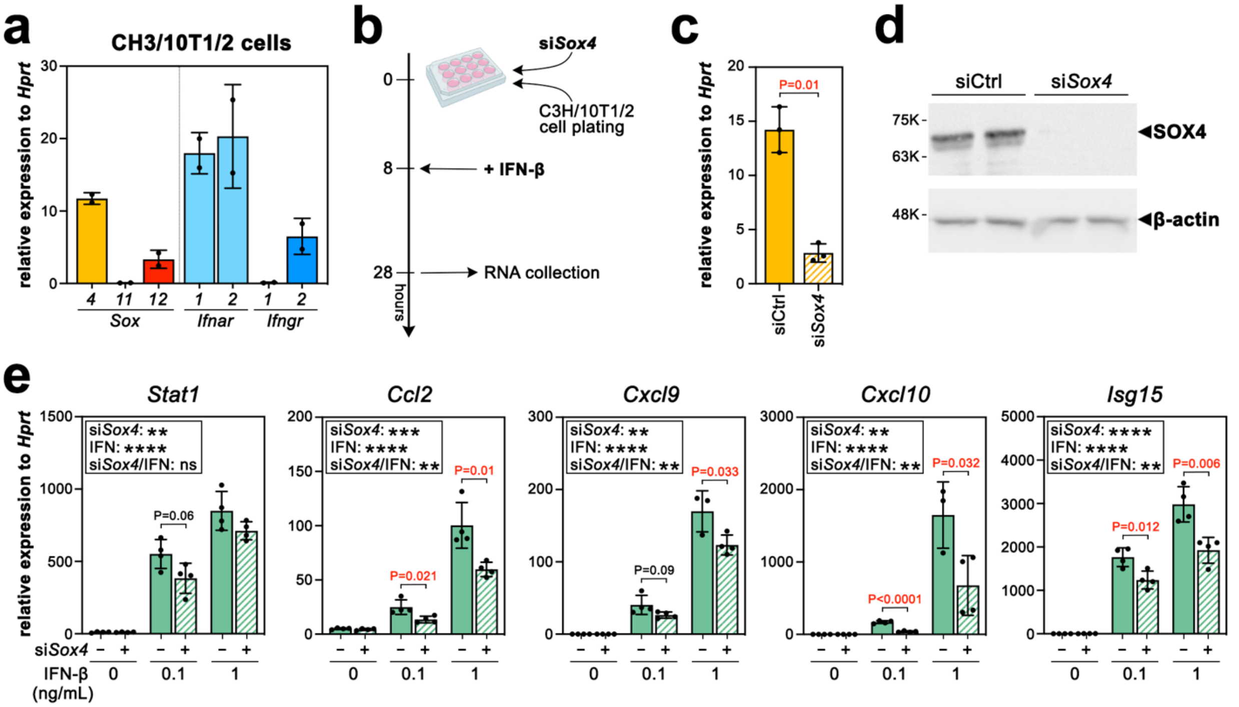

Supplementary figure 10. Effect of *Sox4* knockdown on interferon response in CH3/10T1/2 cells

a. RT-qPCR assay of the expression of genes for SOXC and receptors for type-I (*Ifnar*) and type-II (*Ifngr*) interferons in C3H/10T1/2 cells. Each dot corresponds to an independent replicate. Bars and brackets represent means and standard deviations, respectively.

b. Experimental strategy used to investigate the effect of *Sox4* silencing on the expression of interferon-dependent genes in C3H/10T1/2 cells (image created with BioRender.com).

c. RT-qPCR assay of the expression of *Sox4* in C3H/10T1/2 cells transfected with a control (siCtrl) or a Sox4-specific (si*Sox4*) siRNA for 8 h. Each dot corresponds to an independent replicate. Bars and brackets represent means and standard deviations, respectively. Statistical differences were assessed by two-sided unpaired Student's t-test. P-value ≤0.05 indicates a significant reduction of *Sox4* expression.

d. Western blot assay of the level of SOX4 protein present in C3H/10T1/2 cells transfected for 8 h with a control siRNA (siCtrl) or a *Sox4*-specific siRNA (si*Sox4*). β-actin detection was used as a loading control. The assay was repeated in three independent experiments.

e. RT-qPCR assay of the expression levels of selected interferon-dependent genes in C3H/10T1/2 cells after *Sox4* silencing and IFN-β stimulation. Each dot corresponds to an independent replicate. Bars and brackets represent means and standard deviations, respectively. Statistical differences were assessed by two-sided unpaired Student's t-tests. P-values lower (red) and near (black) 0.05 are indicated. Boxes show the results of two-way ANOVA tests assessing the effects of *Sox4* silencing, IFN-β, and their interaction (ns, non-significant; **, p ≤0.01; ***, p ≤0.001; ****, p ≤0.0001).

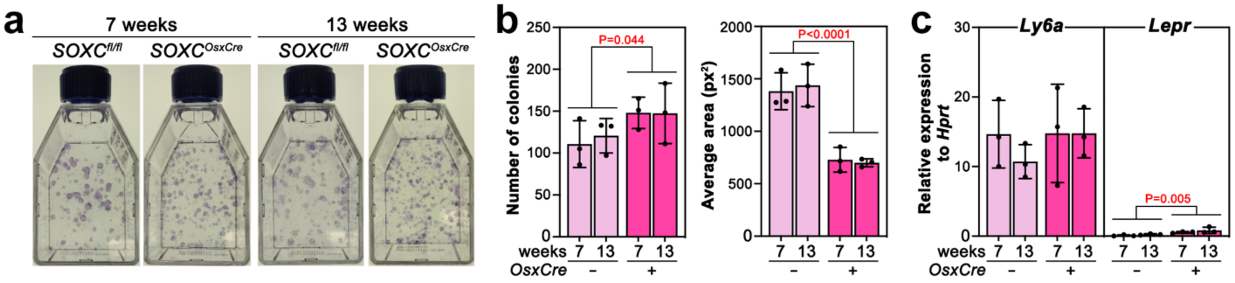

Supplementary figure 11. Properties of SOXC-mutant MSC cultures

a. Representative pictures of CFU-Fs formed by MSCs isolated from the bone marrow of 7- and 13-week-old $SOXC^{OsxCre}$ and control females. Cells were grown for 10 days before staining with methyl violet. The assay was repeated in 6 independent experiments used for analysis in (b).

b. Quantification of the number and average area size of MSC colonies obtained as described in (a). The data suggest that $SOXC^{OsxCre}$ female bone marrow contains more MSCs than controls, but that these cells have a lower proliferation or survival rate. Each dot corresponds to a distinct mouse. Statistical differences were assessed by two-sided unpaired Student's t-tests. P-values ≤0.05 are considered significant.

c. RT-qPCR assay of the expression of *Ly6a* (encoding SCA1) and *Lepr* in MSC colonies obtained as described in (a). The data, presented as in (b), suggest that control and mutant colonies contain primarily $Sca1^{+}$ MSCs.

SUPPLEMENTARY TABLES

Supplementary table 1. PCR primers used to identify *R26*$^{SOX4/+}$ embryonic stem cell clones and genotype mice

PCR primers for ES cell genotyping		
Target	Oligonucleotide sequence	Amplicon size
R26^{WT}	FPa: GAG AAG GGA GCG GAA AAG TCT<br>RPa: CAC CTG TTC AAT TCC CCT GCA	1037 bp
5' *R26*^{SOX4}	FPb: AGG GCG GCT TGG TGC GTT TGC<br>RPb: TGG CAA CTA GAA GGC ACA CTC	1156 and 1377 bp
3' *R26*^{SOX4}	FPc: CAT CGC ATT GTC TGA GTA GGT GTC<br>RPc: AGA GTC TTG CCT GCA AAC CA	3710 bp
**PCR primers for mouse genotyping**		
Target	Oligonucleotide sequence	Amplicon size
Sox4^{+} and *Sox4*^{fl}	FP: GAA GGA GGC GGA GAG TAG ACG G<br>RP: CAT AGC TCAA CAC AAA TGC CAA CGC	450 bp (*Sox4*$^{+}$)<br>520 bp (*Sox4*^{fl})
Sox11^{+} and *Sox11*^{fl}	FP: TTC GTG ATT GAC ACA AAG GCG GAG<br>RP: GCT CCC TGC AGT TTA AGA AAT CGG	319 bp (*Sox11*$^{+}$)<br>467 bp (*Sox11*^{fl})
Sox12^{+} and *Sox12*^{fl}	FP: CCT TCT TGC GCA TGC TTG ATG CTT<br>RP: GGA AAT CAA GTT TCC GGC GAC CAA	324 bp (*Sox12*$^{+}$)<br>435 bp (*Sox12*^{fl})
Cre	FP: TGA GGT TCG CAA GAA CCT GAT GGA<br>RP: GCC GCA TAA CCA GTG AAA CAG CAT	293 bp
R26^{WT}	FP: CTC GTG ATC TGC AAC TCC AGT CTT<br>RP: CCA GAT GAC TAC CTA TCC TCC CAT	210 bp
R26^{SOX4}	FP2: CAT CGC ATT GTC TGA GTA GGT GTC<br>RP: CCA GAT GAC TAC CTA TCC TCC CAT	354 bp

Supplementary table 2. List of primers used in qRT-PCR assays

Gene	Oligonucleotides
Sox4	Forward primer: GCC TCC ATC TTC GTA CAA CC<br>Reverse primer: AGT CAA GCG CGT CTA CCT GT
h*SOX4*	Forward primer: CGA GCT GGG AAT CGC CTC<br>Reverse primer: ATC TGC GAC CAC ACC ATG AA
Mdk	Forward primer: CTT CCG CGA GGG TAC CTG<br>Reverse primer: CCC CCA GCT CTC AAA CTT GTA
Sfrp1	Forward primer: TTC TAC TGG CCC GAG ATG CT<br>Reverse primer: GTT GTC GCA TGG AGG ACA CA
Spp1	Forward primer: AGC TTG GCT TAT GGA CTG AGG<br>Reverse primer: CAG GGA TGA CAT CGA GGG AC

Cst3	Forward primer: GGA GAT GGG CCG AAC TAC AT<br>Reverse primer: GGC ACG CTG TAG ATC TGG AA
Bglap	Forward primer: ACC ATC TTT CTG CTC ACT CTG<br>Reverse primer: GTT CAC TAC CTT ATT GCC CTC C
Runx2	Forward primer: GCT ATT AAA GTG ACA GTG GAC GG<br>Reverse primer: GGC GAT CAG AGA ACA AAC TAG G
Sp7	Forward primer: ATG GCG TCC TCT CTG CTT G<br>Reverse primer: TGA AAG GTC AGC GTA TGG CTT
Col1a1	Forward primer: GCT CCT CTT AGG GGC CAC T<br>Reverse primer: CCA CGT CTC ACC ATT GGG G
Col3a1	Forward primer: TTC TTC TCA CCC TTC TTC ATC C<br>Reverse primer: TCT CTA GAC TCA TAG GAC TGA CC
Postn	Forward primer: AGT CAT TCA AGG CAG TCT TCA<br>Reverse primer: TTC GCC TTC TTT AAT CAG CCT
Stat1	Forward primer: GCC GAG AAC ATA CCA GAG AAT C<br>Reverse primer: GAT GTA TCC AGT TCG CTT AGG G
Isg15	Forward primer: ACC TAG AGC TAG AGC CTG C<br>Reverse primer: TTC AGT TCT GAC ACC GTC ATG
Ccl2	Forward primer: GTC CCT GTC ATG CTT CTG G<br>Reverse primer: GCT CTC CAG CCT ACT CAT TG
Cxcl9	Forward primer: CAC GAT CCA CTA CAA ATC CCT C<br>Reverse primer: TCT TCA CAT TTG CCG AGT CC
Cxcl10	Forward primer: TCA GCA CCA TGA ACC CAA G<br>Reverse primer: CTA TGG CCC TCA TTC TCA CTG
Hprt	Forward primer: CCT CAT GGA CTG ATT ATG GAC AG<br>Reverse primer: TCA GCA AAG AAC TTA TAG CCC C
