# Supplementary material for: SOXC are critical regulators of adult bone mass
Source: Nat Commun. 2024 Apr 5;15:2956. doi: 10.1038/s41467-024-47413-2 (PMC10997656; doi:10.1038/s41467-024-47413-2)
Supplement: Supplementary file 3 — Description of Additional Supplementary Files [file 41467_2024_47413_MOESM3_ESM.pdf]

Description of Additional Supplementary Files:

Supplementary Data 1: Lists of the marker genes of each cell population identified in scRNA-seq assays.

Supplementary Data 2: Lists of genes differentially expressed in SOXCOsxCre versus control cell populations identified in scRNA-seq assays.

Supplementary Data 3: Lists of biological processes identified by Gene Ontology analysis that involve genes differentially expressed in SOXCOsxCre versus control cell populations.

Supplementary Data 4: Lists of regulons affected in SOXCOsxCre versus control cell populations and lists of SOX4 target genes predicted by SCENIC analysis.
