# Supplementary material for: SOXC are critical regulators of adult bone mass
Source: Nat Commun. 2024 Apr 5;15:2956. doi: 10.1038/s41467-024-47413-2 (PMC10997656; doi:10.1038/s41467-024-47413-2)
Supplement: Supplementary file 8 — Reporting Summary [file 41467_2024_47413_MOESM8_ESM.pdf]

nature portfolio

Corresponding author(s): Véronique Lefebvre and Marco Angelozzi

Last updated by author(s): Dec 21, 2023

Reporting Summary

Nature Portfolio wishes to improve the reproducibility of the work that we publish. This form provides structure for consistency and transparency in reporting. For further information on Nature Portfolio policies, see our Editorial Policies and the Editorial Policy Checklist.

Statistics

For all statistical analyses, confirm that the following items are present in the figure legend, table legend, main text, or Methods section.

n/a	Confirmed	
☐	☒	The exact sample size (*n*) for each experimental group/condition, given as a discrete number and unit of measurement
☐	☒	A statement on whether measurements were taken from distinct samples or whether the same sample was measured repeatedly
☐	☒	The statistical test(s) used AND whether they are one- or two-sided<br>*Only common tests should be described solely by name; describe more complex techniques in the Methods section.*
☐	☒	A description of all covariates tested
☐	☒	A description of any assumptions or corrections, such as tests of normality and adjustment for multiple comparisons
☐	☒	A full description of the statistical parameters including central tendency (e.g. means) or other basic estimates (e.g. regression coefficient) AND variation (e.g. standard deviation) or associated estimates of uncertainty (e.g. confidence intervals)
☐	☒	For null hypothesis testing, the test statistic (e.g. *F*, *t*, *r*) with confidence intervals, effect sizes, degrees of freedom and *P* value noted<br>*Give P values as exact values whenever suitable.*
☒	☐	For Bayesian analysis, information on the choice of priors and Markov chain Monte Carlo settings
☒	☐	For hierarchical and complex designs, identification of the appropriate level for tests and full reporting of outcomes
☒	☐	Estimates of effect sizes (e.g. Cohen's *d*, Pearson's *r*), indicating how they were calculated

Our web collection on statistics for biologists contains articles on many of the points above.

Software and code

Policy information about availability of computer code

Data collection	- µCT images were acquired with a built-in software in a SCANCO µCT 45 scanner (SCANCO Medical AG) or an eXplore Locus SP scanner (Trifoil Imaging);<br>- Radiographic images were acquired using a built-in software in Faxitron X-ray system (Hologic);<br>- Histology and in situ assay images were acquired with a ZEN2.6 software (blue edition) in Axio Scan.Z1 scanner (ZEISS) or a Leica LAS-X software in Leica TCS SP8 confocal microscope (Leica Microsystems);<br>- Western blot images were acquired using a built-in software in a ChemiDoc Gel Imaging System (Bio-Rad Laboratories);<br>- Gene expression data by RT-qPCR were generated using QuantStudio Design & Analysis software v4.1.3 in a QuantStudio 3 system (Thermo Fisher Scientific).
Data analysis	- µCT images were analyzed with a µCT Evaluation Program v6.6 (SCANCO Medical AG) or MicroView v. 2.2 (GE Healthcare);<br>- Radiographic images were analyzed using a built-in software in Faxitron X-ray system (Hologic);<br>- Bone histomorphometry analysis were performed with the BIOQUANT OSTEO software (BIOQUANT);<br>- Single-cell RNA-sequencing data were demultiplexed and aligned to the mouse genome reference with Cell Ranger v6.1.2 (10X Genomics). Further analysis were performed in R v4.3.2 or python v3.9 using the following packages: Seurat v4; velocyto.R v0.6; scVelo v0.2.5; cellDancer v0.2.5; slingshot v2.10; condiments v.1.10; SCENIC v1.3.1;<br>- Images were analyzed with ImageJ 1.53a (NIH) or AdobePhotoshop v24.5.0 (Adobe);<br>- Graphical representation and statistical analysis were performed using GraphPad Prism v10.0.1 (GraphPad).

For manuscripts utilizing custom algorithms or software that are central to the research but not yet described in published literature, software must be made available to editors and reviewers. We strongly encourage code deposition in a community repository (e.g. GitHub). See the Nature Portfolio guidelines for submitting code & software for further information.

Data

Policy information about availability of data

All manuscripts must include a data availability statement. This statement should provide the following information, where applicable:

- Accession codes, unique identifiers, or web links for publicly available datasets
- A description of any restrictions on data availability
- For clinical datasets or third party data, please ensure that the statement adheres to our policy

The scRNA-seq data generated in this study are available at the NCBI Gene Expression Omnibus repository (accession number GSE241637).

Research involving human participants, their data, or biological material

Policy information about studies with human participants or human data. See also policy information about sex, gender (identity/presentation), and sexual orientation and race, ethnicity and racism.

Reporting on sex and gender	*Use the terms sex (biological attribute) and gender (shaped by social and cultural circumstances) carefully in order to avoid confusing both terms. Indicate if findings apply to only one sex or gender; describe whether sex and gender were considered in study design; whether sex and/or gender was determined based on self-reporting or assigned and methods used. Provide in the source data disaggregated sex and gender data, where this information has been collected, and if consent has been obtained for sharing of individual-level data; provide overall numbers in this Reporting Summary. Please state if this information has not been collected. Report sex- and gender-based analyses where performed, justify reasons for lack of sex- and gender-based analysis.*
Reporting on race, ethnicity, or other socially relevant groupings	*Please specify the socially constructed or socially relevant categorization variable(s) used in your manuscript and explain why they were used. Please note that such variables should not be used as proxies for other socially constructed/relevant variables (for example, race or ethnicity should not be used as a proxy for socioeconomic status). Provide clear definitions of the relevant terms used, how they were provided (by the participants/respondents, the researchers, or third parties), and the method(s) used to classify people into the different categories (e.g. self-report, census or administrative data, social media data, etc.) Please provide details about how you controlled for confounding variables in your analyses.*
Population characteristics	*Describe the covariate-relevant population characteristics of the human research participants (e.g. age, genotypic information, past and current diagnosis and treatment categories). If you filled out the behavioural & social sciences study design questions and have nothing to add here, write "See above."*
Recruitment	*Describe how participants were recruited. Outline any potential self-selection bias or other biases that may be present and how these are likely to impact results.*
Ethics oversight	*Identify the organization(s) that approved the study protocol.*

Note that full information on the approval of the study protocol must also be provided in the manuscript.

Field-specific reporting

Please select the one below that is the best fit for your research. If you are not sure, read the appropriate sections before making your selection.

[x] Life sciences [ ] Behavioural & social sciences [ ] Ecological, evolutionary & environmental sciences

For a reference copy of the document with all sections, see nature.com/documents/nr-reporting-summary-flat.pdf

Life sciences study design

All studies must disclose on these points even when the disclosure is negative.

Sample size	≥3 samples were used for each experimental group. Sample size was determined using results obtained from preliminary experiments and previous published studies in the same research field.
Data exclusions	Mice with health conditions unrelated to their genotype were excluded from the study.
Replication	For in vivo assays we used ≥3 mice per group. In vitro experiments were performed at least twice with technical triplicates to ensure reproducibility.
Randomization	Experiments were not randomized since mice were assigned to experimental groups based on genotype and sex. All mice were kept on an enriched C57B6/J background and housed in standardized conditions to avoid variation among groups.
Blinding	µCT and histomorphometry analysis were conducted blinded using unique sample identifier which did not contain information about sex, age or genotype of the mouse. However, it is important to notice that blinding may been have irrelevant in some group analysis due to the strong phenotypic difference between control and mutant mice.

Reporting for specific materials, systems and methods

We require information from authors about some types of materials, experimental systems and methods used in many studies. Here, indicate whether each material, system or method listed is relevant to your study. If you are not sure if a list item applies to your research, read the appropriate section before selecting a response.

Materials & experimental systems

n/a	Involved in the study
☐	☒ Antibodies
☐	☒ Eukaryotic cell lines
☒	☐ Palaeontology and archaeology
☐	☒ Animals and other organisms
☒	☐ Clinical data
☒	☐ Dual use research of concern
☒	☐ Plants

Methods

n/a	Involved in the study
☒	☐ ChIP-seq
☒	☐ Flow cytometry
☒	☐ MRI-based neuroimaging

Antibodies

Antibodies used	- SOX4 antibody; MA5-31423, Thermo Fisher Scientific; 1:500 for immunostaining and 1:2000 for Western blot assay.<br>- anti-mouse IgG2b antibody, biotinylated; ab97248, Abcam; 1:3000 for immunostaining.<br>- β-actin antibody; SC47778, Santa Cruz Biotechnology; 1:2000 for Western blot assay.<br>- anti-mouse antibody, peroxidase-conjugated; 1706516, Bio-Rad Laboratories; 1:5000 for Western blot assay.<br>- FLAG antibody, peroxidase-conjugated; A8592, Sigma-Aldrich; 1:10000 for Western blot assay.<br>- mouse lineage cell depletion kit; 130-090-858, Miltenyi Biotec; used according to manufacturer's instruction for cell sorting.<br>- TER-119 antibody, biotinylated; 13-5921-85, Thermo Fisher Scientific; 10 μL per sample for cell sorting.<br>- CD71 antibody, biotinylated; 1130-109-572, Miltenyi Biotec; 20 μL per sample for cell sorting.<br>- CD3e antibody, biotinylated; 130-093-179, Miltenyi Biotec, 10 μL per sample for cell sorting.<br>- anti-CD45 microbeads; 130-052-301, Miltenyi Biotec; used according to manufacturer's instruction for cell sorting.
Validation	All antibodies were tested in preliminary experiments with appropriate controls before use.<br>For immunostaining, antibodies were tested on tissues and cells known to express the protein, while a non-specific IgG antibody was used as negative control.<br>For Western blot assays, lysates from cells overexpressing or silenced for the expression of the protein were used as positive and negative controls, respectively.<br>Efficiency of antibodies used in cell sorting experiments was tested by following FACS analysis and RT-qPCR assays for the expression of markers for the populations expected to be depleted or enriched in the procedure.

Eukaryotic cell lines

Policy information about cell lines and Sex and Gender in Research

Cell line source(s)	C3H/10T1/2 cells, Clone 8 obtained from ATCC (cat. #CCL-226).<br>Primary mesenchymal stem cells (MSCs) were obtained from the bone marrow of 7- and 13-week-old female mice as described in the Methods section.
Authentication	C3H/10T1/2 cells were freshly obtained from ATCC.<br>Identity of primary MSCs was confirmed by RT-qPCR analysis for the expression of typical markers (i.e., Sca1, Pdgfra, Cd51, Lepr).
Mycoplasma contamination	Mycoplasma tests (MycoAlert® Mycoplasma Detection Kit; LT07-218; Lonza) were routinely performed on cell media to confirm the absence of Mycoplasma in cell cultures.
Commonly misidentified lines<br>(See ICLAC register)	No commonly misidentified cell lines were used.

Animals and other research organisms

Policy information about studies involving animals; ARRIVE guidelines recommended for reporting animal research, and Sex and Gender in Research

Laboratory animals	Mouse lines used in this study were kept on an enriched C57B6/J genetic background. Mice were used between 5 and 52 weeks of age.
Wild animals	No wild animals were used in this study.

Reporting on sex	Except for single-cell RNA-seq analysis, all experiments were performed on both male and female mice separately as shown throughout the manuscript.
Field-collected samples	This study did not involve samples collected from the field.
Ethics oversight	Mice were used as approved by the Cleveland Clinic (IAC 2017–1867) and Children's Hospital of Philadelphia Institutional Animal Care and Use Committees (IAC 21-001297).

Note that full information on the approval of the study protocol must also be provided in the manuscript.

Plants

Seed stocks	*Report on the source of all seed stocks or other plant material used. If applicable, state the seed stock centre and catalogue number. If plant specimens were collected from the field, describe the collection location, date and sampling procedures.*
Novel plant genotypes	*Describe the methods by which all novel plant genotypes were produced. This includes those generated by transgenic approaches, gene editing, chemical/radiation-based mutagenesis and hybridization. For transgenic lines, describe the transformation method, the number of independent lines analyzed and the generation upon which experiments were performed. For gene-edited lines, describe the editor used, the endogenous sequence targeted for editing, the targeting guide RNA sequence (if applicable) and how the editor was applied.*
Authentication	*Describe any authentication procedures for each seed stock used or novel genotype generated. Describe any experiments used to assess the effect of a mutation and, where applicable, how potential secondary effects (e.g. second site T-DNA insertions, mosiacism, off-target gene editing) were examined.*
